# K-Ras Activation Induces Differential Sensitivity to Sulfur Amino Acid Limitation and Deprivation and to Oxidative and Anti-Oxidative Stress in Mouse Fibroblasts

**DOI:** 10.1371/journal.pone.0163790

**Published:** 2016-09-29

**Authors:** Gaia De Sanctis, Michela Spinelli, Marco Vanoni, Elena Sacco

**Affiliations:** 1 SYSBIO, Centre of Systems Biology, Milan, Italy; 2 Department of Biotechnology and Biosciences, University of Milano-Bicocca, Piazza della Scienza 2, 20126, Milan, Italy; National Institute for Agronomic Research, FRANCE

## Abstract

**Background:**

Cancer cells have an increased demand for amino acids and require transport even of non-essential amino acids to support their increased proliferation rate. Besides their major role as protein synthesis precursors, the two proteinogenic sulfur-containing amino acids, methionine and cysteine, play specific biological functions. In humans, methionine is essential for cell growth and development and may act as a precursor for cysteine synthesis. Cysteine is a precursor for the biosynthesis of glutathione, the major scavenger for reactive oxygen species.

**Methodology and Principal Findings:**

We study the effect of *K-ras* oncogene activation in NIH3T3 mouse fibroblasts on transport and metabolism of cysteine and methionine. We show that cysteine limitation and deprivation cause apoptotic cell death (cytotoxic effect) in both normal and *K*-*ras*-transformed fibroblasts, due to accumulation of reactive oxygen species and a decrease in reduced glutathione. Anti-oxidants glutathione and MitoTEMPO inhibit apoptosis, but only cysteine-containing glutathione partially rescues the cell growth defect induced by limiting cysteine. Methionine limitation and deprivation has a cytostatic effect on mouse fibroblasts, unaffected by glutathione. *K*-*ras*-transformed cells–but not their parental NIH3T3—are extremely sensitive to methionine limitation. This fragility correlates with decreased expression of the *Slc6a15* gene—encoding the nutrient transporter SBAT1, known to exhibit a strong preference for methionine—and decreased methionine uptake.

**Conclusions and Significance:**

Overall, limitation of sulfur-containing amino acids results in a more dramatic perturbation of the oxido-reductive balance in *K-ras*-transformed cells compared to NIH3T3 cells. Growth defects induced by cysteine limitation in mouse fibroblasts are largely–though not exclusively–due to cysteine utilization in the synthesis of glutathione, mouse fibroblasts requiring an exogenous cysteine source for protein synthesis. Therapeutic regimens of cancer involving modulation of methionine metabolism could be more effective in cells with limited methionine transport capability.

## Introduction

Activation of the *K-ras* proto-oncogene [1,2,3,4] has a great incidence in human tumors, as reported in the catalogue of somatic mutations in cancer (COSMIC) [5]. *K-ras* activation occurs in 22% of all tumors, prevalently in pancreatic carcinomas (about 90%), colorectal carcinomas (40–50%), and lung carcinomas (30–50%), as well as in biliary tract malignancies, endometrial cancer, cervical cancer, bladder cancer, liver cancer, myeloid leukemia and breast cancer. K-Ras oncoproteins are important clinical targets for anti-cancer therapy [6] and several strategies have been explored in order to inhibit aberrant Ras signaling, as reviewed in [7,8,9,10].

The acquisition of important hallmark traits of cancer cells, including enhanced cell growth and survival, rely on deep changes in metabolism driven by oncogene activation [11,12,13,14,15]. Oncogenic activation of *K-ras* contributes to the acquisition of the hyper-glycolytic phenotype (also known as Warburg effect, from the pioneering studies of Warburg [16]) due to enhancement in glucose transport and aerobic glycolysis [17,18]. *K-ras* oncogene activation also correlates with down-regulated expression of mitochondrial genes, altered mitochondrial morphology and production of large amount of reactive oxygen species (ROS) associated with mitochondrial metabolism [19,20]. Furthermore, *K-ras* activation allows cells to make extensive anaplerotic usage of glutamine, the more concentrated amino acid in human plasma [21]. In Ras-transformed cells, glutamine is largely utilized through reductive carboxylation that results in a non-canonical tricarboxylic acid cycle (TCA) pathway [19,22,23,24,25,26]. These metabolic changes render Ras-transformed cells addicted to glutamine, and to glutaminolysis, and offer new therapeutic opportunities. Indeed, glutamine metabolism restriction and targeted cancer therapeutics directed against glutamine transporters or glutaminolysis can be used to limit tumor cell proliferation and survival without affecting normal cells [27,28,29].

Besides glutamine transporters, all amino acid transporters are being receiving attention from scientific community as potential drug targets for cancer treatment, given the increased demand of cancer cells for these nutrients to support their enhanced cell growth [30,31]. Selective blockers of these transporters might be effective in preventing the entry of important amino acids into tumor cells, thus essentially starving these cells to death.

Methionine is an essential amino acid required for normal growth and development in mammals [32]. The intracellular level of methionine depends on the balance between synthesis (through the *de novo* synthetic pathway), recycle (through the *salvage* pathway), consumption (in biosynthesis of proteins) and its transport. An important metabolite of methionine is S-adenosylmethionine (SAM), the principal methyl donor in the cell. SAM is required for methylation of DNA, RNA, proteins (including histones [33]) and lipids by the enzymes methyltransferases. Moreover, SAM is involved in biosynthesis of polyamines, which have far-ranging effects on nuclear and cell division, and methionine salvage pathway [34]. SAM gives its activated methyl group in methylation reactions, being converted to S-adenosylhomocysteine, which is reversibly hydrolyzed to homocysteine (S1 Fig). Depending on demand, homocysteine metabolism can be either directed toward the re-methylation pathway to regenerate methionine (thus increasing methylation potential) or toward antioxidant synthesis in the trans-sulfuration pathway [34]. In the first catabolic step of trans-sulfuration, homocysteine may be condensed to serine to form cystathionine, which in turn may be converted to cysteine [35].

Cysteine is a sulfur-containing, semi-essential proteinogenic amino acid. It can be synthesized in humans to some extent; as such, it is classified as conditionally essential, since it may become temporarily essential when synthesis during rapid growth or critical illness is insufficient [36]. Cysteine is a precursor for the tripeptide glutathione, an important intracellular antioxidant that reduces reactive oxygen species (ROS), thereby protecting cells from oxidative stress [37]. The systemic availability of oral glutathione (GSH) is negligible; so it must be biosynthesized from its constituent amino acids, cysteine, glycine, and glutamic acid, the first being the limiting substrate [38]. Furthermore, cysteine is a precursor for the production of taurine, another antioxidant, and sulfate [39]. At least in liver, glutathione also acts as cysteine storage, from which this amino acid can be mobilized if required to maintain protein synthesis under nutritional stress [40]. Under normal physiological conditions, cysteine can usually be synthesized *de novo* from homocysteine in humans if a sufficient quantity of methionine is available.

Normal mouse fibroblasts (NIH3T3) and their derived cells stably expressing oncogenic K-*ras* mutant (NIH-RAS) proved to be a valid cellular model for studying Ras-dependent transcriptional reprogramming [41] and metabolic rewiring [23,42,43]. The Ras-dependent transformation phenotypes of NIH-RAS cells can be down-regulated by over-expressing a dominant negative mutant of RasGRF1 with Ras sequestering properties, extensively characterized in our laboratory [7,44,45]. We use these cell lines to study the effect of *K-ras* proto-oncogene activation on transport and metabolism of the proteinogenic sulfur amino acids, cysteine and methionine.

We show that cysteine limitation and deprivation increase ROS level and decrease reduced glutathione, eventually leading to apoptotic cell death. Through the complementary use of anti-oxidants glutathione and MitoTEMPO (a cysteine non-containing reducing agent) and inhibitors of *de novo* biosynthesis of reduced glutathione, we show that growth defects induced by cysteine limitation in mouse fibroblasts are largely–though not exclusively–due to cysteine utilization in the synthesis of glutathione and that mouse fibroblasts require an exogenous cysteine source for protein synthesis. Methionine limitation and deprivation is cytostatic and unaffected by glutathione. Limitation of sulfur-containing amino acids perturbs the oxidoreductive balance, particularly in *K-ras*-transformed cells that display selective growth fragility to a moderate reduction in methionine supply. Such nutritional fragility correlates with Ras activation, decreased expression of the *Slc6a15* gene -encoding the methionine transporter SBAT1- and reduced methionine uptake.

## Results

### Methionine limitation reduces growth of Ras-transformed mouse fibroblasts more than growth of normal cells

**Fig 1:**
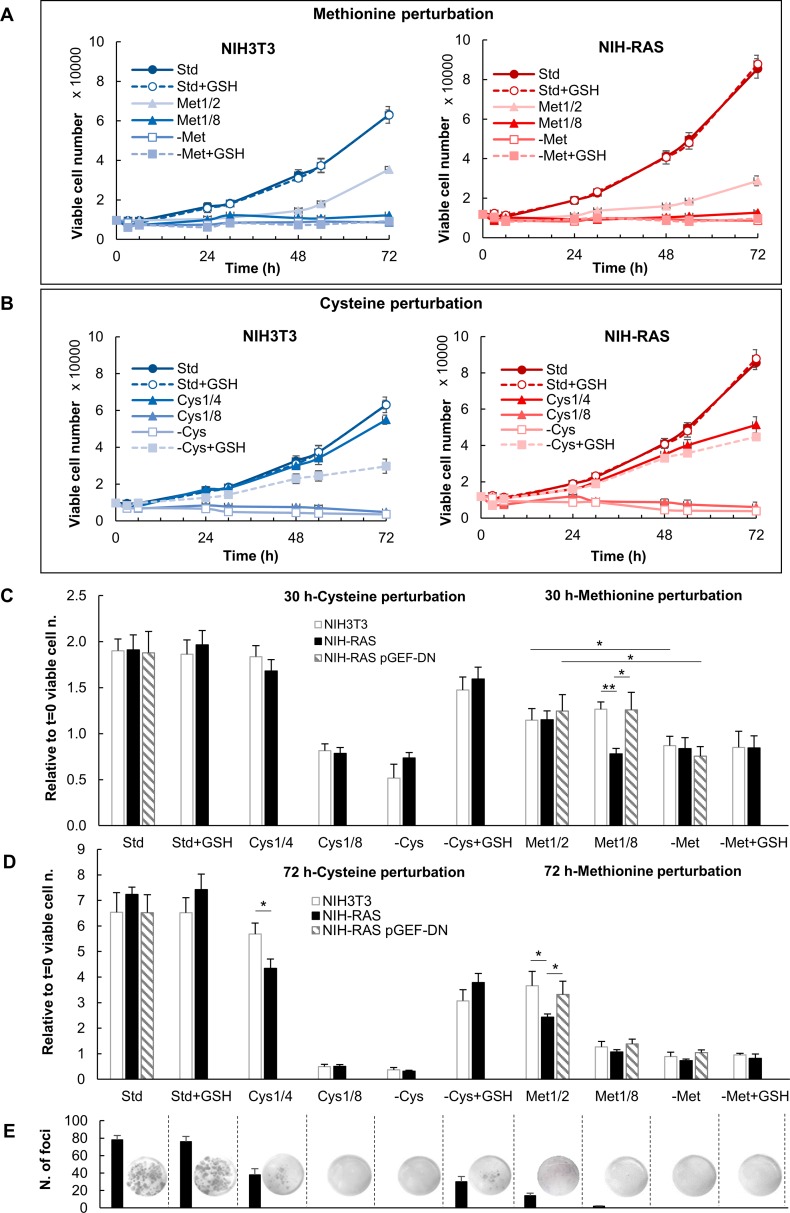
Proliferation under methionine and cysteine deprivation and limitation. Cell proliferation of NIH3T3 and NIH-RAS cells grown in media supplemented with different concentrations of methionine and glutathione (A) or cysteine and glutathione (B) and counted daily for 72 h of growth under conditions indicated. Plotted data are mean +/- standard deviation computed from at least three independent experiments. (C-D) Cell proliferation of NIH3T3, NIH-RAS and NIH-RAS pGEF-DN cells grown for 30 h (C) and 72 h (D) under conditions indicated. (E) *Foci* formation of NIH-RAS cells grown for 9 days under conditions indicated. *P<0.05; **P<0.01 (Student’s *t*-test).

First, we analyzed cell proliferation of normal NIH3T3 and Ras-transformed NIH-RAS mouse fibroblasts under standard growth condition (0.2 mM Cys, 0.2 mM Met), limitation (1/8: 0.025 mM; 1/4: 0.05 mM; 1/2: 0.1 mM) and deprivation of cysteine or methionine. Both cell lines were unable to grow in the absence of either methionine or cysteine (Fig 1A and 1B, open squares), demonstrating that both sulfur amino acids are essential for cell proliferation of mouse fibroblasts, which are not able to synthesize neither cysteine nor methionine each from the other.

Growth of NIH-RAS cells was more severely inhibited by methionine limitation than that of NIH3T3 cells. In Met_1/2_ condition (Fig 1A and 1B, light triangles, and Fig 1D) the mass duplication time (MTD) of NIH-RAS cells was 1.5 longer than that of NIH3T3 (S1 Table). More stringent methionine limitation (Met_1/8_) resulted in almost complete arrest of cell proliferation of both cell lines (Fig 1A, dark filled triangles, and S1 Table). Fig 1C and 1D show cell proliferation data 30 and 72 hours after methionine limitation, highlighting enhanced sensitivity of transformed NIH3T3 cells to methionine limitation. Note that NIH-RAS cells grown in Met_1/2_ condition are still largely viable after 72 hours, unlike the cells grown in Met_1/8_ condition (Fig 1D). Under methionine limitation NIH-RAS cells were also severely hampered in *foci* formation ability (Fig 1E). Notably the major sensitivity of Ras-transformed cells to methionine limitation was fully reverted by the over-expression of a dominant negative mutant of the Ras-specific guanine nucleotide exchange factor RasGRF1 (RasGRF1^W1056E^, that we refer to as GEF-DN), endowed with Ras sequestering properties (Fig 1C and 1D, S2 and S3 Figs).

All together, these data indicate that Ras hyper-activation enhances sensitivity to methionine limitation in mouse fibroblasts.

As shown in Fig 1B and in S1 Table the cell proliferation behavior under cysteine limitation and deprivation was quite similar in NIH3T3 and NIH-RAS cells. In Cys_1/2_ condition both cell lines grew as well as in standard medium (S4A Fig, S1 Table), while further reduction of cysteine (Cys_1/4_) increased the MDT of both NIH3T3 and NIH-RAS cells, even if slightly more in transformed cells (Fig 1B, S1 Table). Cysteine limitation strongly reduced *foci* formation ability of NIH-RAS cells (Fig 1E).

### Cysteine mainly acts as a precursor of glutathione, whose excess mostly affects normal cells

**Fig 2:**
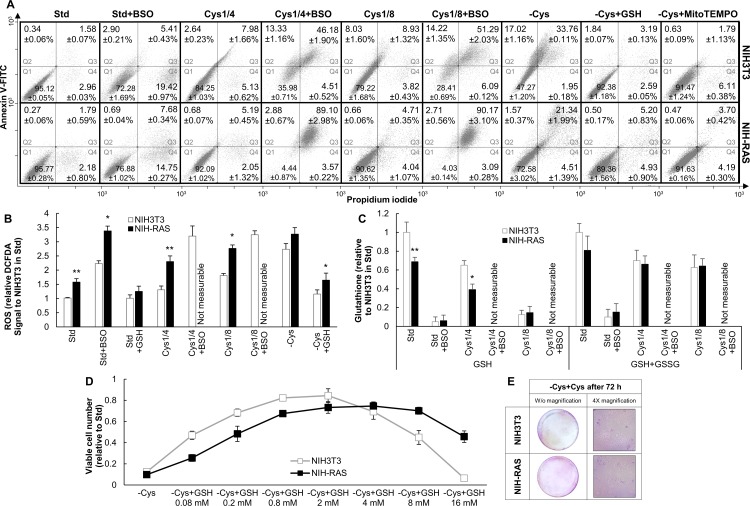
Viability and redox state under cysteine deprivation and limitation. (A) Representative dot plots for NIH3T3 and NIH-RAS cells stained with Annexin V-FITC and propidium iodide and analyzed by FACS after 30 h of growth under conditions indicated. Q1 = quadrant 1, healthy cell; Q2 = quadrant 2, early apoptotic cells; Q3 = quadrant 3, late apoptotic cells; Q4 = quadrant 4, necrotic cells. MitoTEMPO and buthionine sulfoximine (BSO) were used at the concentration of 10 μM and 100 μM, respectively. The values reported for each quadrant are the mean +/- standard deviation of three independent experiments. (B) Relative ROS levels in NIH3T3 and NIH-RAS cells grown for 48 h under conditions indicated as determined by DCFDA (2’,7’-dichlorodihydrofluorescein diacetate) staining. Each bar represents the mean of at least three independent experiments with error bars representing the standard deviation. (C) Reduced and total glutathione levels (measured as described in [55]) in NIH3T3 and NIH-RAS cells grown for 48 h under conditions indicated. Each bar represents the mean of at least three independent experiments with error bars representing the standard deviation. (D) Cell proliferation of NIH3T3 and NIH-RAS cells grown for 48 h in cysteine-free medium supplemented with different concentrations of glutathione. Plotted data are mean +/- standard deviation computed from at least three independent experiments. *P<0.05; **P<0.01 (Student’s *t*-test). (E) Crystal violet staining of NIH3T3 and NIH-RAS cells plated at the density of 9000 cells/cm^2^, grown for 72 h under cysteine deprivation and then for 48 h in standard medium.

Apoptotic and necrotic cell death can be assayed by FACS after staining with Annexin V-FITC and propidium iodide (PI). After limitation or deprivation of cysteine for 30 hours, apoptotic cells significantly increased in both cell lines, the effect being stronger in cysteine-deprived cells (Fig 2A). Supplementation of cysteine to cells grown for 72 hours in cysteine-free medium did not result in any significant growth recovery, reinforcing the notion that cysteine deprivation exerted a cytotoxic effect (cell death) in both NIH3T3 and NIH-RAS cell lines (Fig 2E).

Glutathione is the most important endogenous antioxidant in mammalian cells, and the major redox buffer responsible for redox homeostasis [46,47]. It acts as a ROS scavenger through its oxidation to GSSG. The reduced form (GSH) is restored at the expenses of NADPH. The intracellular concentration of GSH depends on a dynamic balance between synthesis, consumption rate (metabolism), and its transport.

We measured ROS (by FACS analysis of DCFDA-stained cells, Fig 2B), endogenous total (GSH+GSSG) and reduced (GSH) glutathione levels (by an enzymatic assay, Fig 2C) in NIH3T3 and NIH-RAS cells in standard medium and 48 h after perturbing cysteine metabolism. In keeping with literature data [19,45,48,49], in standard medium NIH-RAS cells showed a 1.7-fold higher ROS level than NIH3T3 (Fig 2B), accompanied by a moderate decrease in total glutathione and a significant decrease in reduced glutathione (Fig 2C). Cysteine limitation and deprivation induced an increase in ROS levels, the effect being stronger in NIH-RAS cells (Fig 2B). Under cysteine limitation, total glutathione levels (GSH+GSSG) were lower than in standard condition (Fig 2C), consistently with the notion that cysteine availability is rate-limiting for GSH synthesis [50,51,52,53]. The high cell mortality under cysteine deprivation hindered the measurement of glutathione levels.

To investigate whether mouse fibroblasts are dependent on cysteine for growth, or whether the growth defects are the result of the oxidative stress on the cells, we took two complementary approaches. First, we modulated the oxidative response of cysteine-depleted cells with either cysteine-containing (GSH) or cysteine-non-containing (MitoTEMPO) anti-oxidants. Second, we blocked glutathione *de novo* biosynthesis of standard or cysteine-limited cells with buthionine sulfoximine (BSO), that blocks the activity of gamma-glutamylcysteine synthetase (γ-GCT) required for the formation of the glutathione precursor gamma-glutamylcysteine from glutamate and cysteine [47].

Supplementing GSH to cysteine-free medium (-Cys+GSH growth condition) partially restored cell proliferation (Fig 1A, 1C and 1D) in both NIH3T3 and NIH-RAS cell lines and the ability to form *foci* in NIH-RAS cells (Fig 1E). Also, supplementation of GSH significantly reduced apoptosis induced by cysteine withdrawal and fully restored ROS levels to basal (Fig 2A and 2B). Compared to NIH3T3, NIH-RAS cells require a higher GSH concentration both to recover cell survival and growth as well as to show the “anti-oxidative stress” (Fig 2D), phenomenon described in [54]. The reduced sensitivity to both positive and negative effects of GSH is most likely the result of the lower GSH content of Ras-transformed cells (Fig 2C). Supplementation of MitoTEMPO in cysteine-free medium reduced apoptosis to the same levels observed after GSH addition (Fig 2A), but could not rescue cell proliferation under cysteine deprivation (S4B Fig).

In both NIH3T3 and NIH-RAS cell lines, BSO treatment severely down-regulated glutathione accumulation (Fig 2C) and reduced proliferation (S4C Fig). Concurrently, both ROS accumulation (Fig 2B) and the fraction of apoptotic cells increased (Fig 2A). These effects appear stronger in NIH-RAS than in NIH3T3 cells. They are dramatically enhanced by growth in limiting cysteine, which results in the death of most cells within 30 h from the treatment (Fig 2A). Cell death in BSO-treated cells grown in the absence of cysteine was essentially caused by oxidative stress, since almost all cells were strongly positive to DCFDA staining, as shown by fluorescence-microscopy analysis (S4D Fig). In these conditions ca 90% and 50% of NIH-RAS and NIH3T3 cells, respectively, are apoptotic after 30 h of treatment (Fig 2A). All together, these data confirm the major dependence of NIH-RAS from cysteine availability for the maintenance of proper GSH levels, redox homeostasis and cell viability, and on the other hand suggest that NIH3T3 cells less recur to the *de novo* synthesis of GSH to maintain redox homeostasis and favorable growth conditions.

### Ras-transformed mouse fibroblasts show lower expression of a gene encoding a methionine-transporting solute carrier and reduced methionine uptake than normal cells

**Fig 3:**
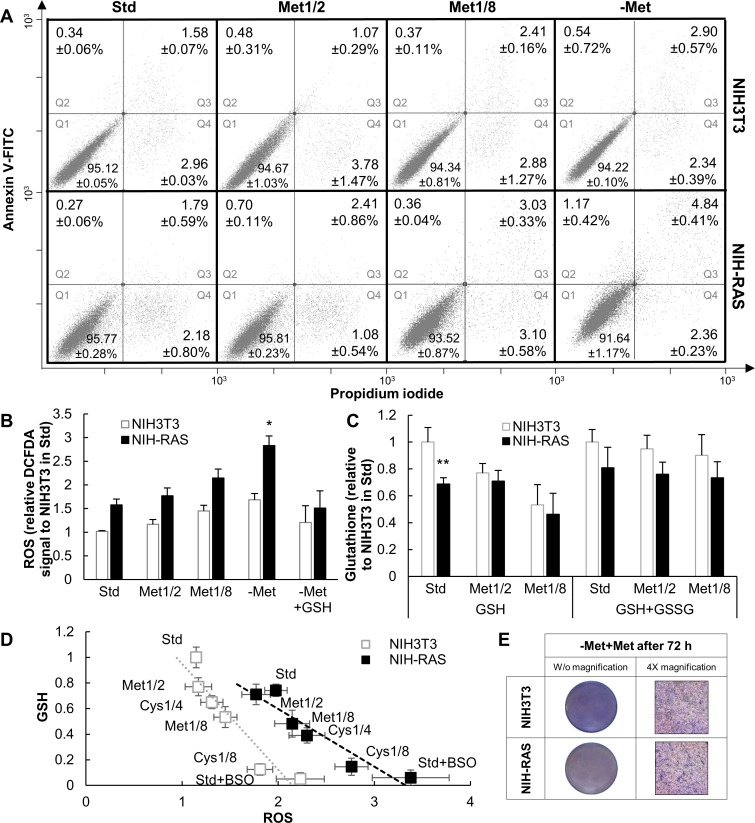
Viability and redox state under methionine deprivation and limitation. (A) Representative dot plots for NIH3T3 and NIH-RAS cells stained with Annexin V-FITC and propidium iodide and analyzed by FACS after 30 h of growth under conditions indicated. Q1 = quadrant 1, healthy cell; Q2 = quadrant 2, early apoptotic cells; Q3 = quadrant 3, late apoptotic cells; Q4 = quadrant 4, necrotic cells. The values reported for each quadrant are the mean +/- standard deviation of three independent experiments. (B) Relative ROS levels in NIH3T3 and NIH-RAS cells grown for 48 h under conditions indicated as determined by DCFDA (2’,7’-dichlorodihydrofluorescein diacetate) staining. Each bar represents the mean of at least three independent experiments with error bars representing the standard deviation. (C) Reduced and total glutathione levels (measured as described in [55]) in NIH3T3 and NIH-RAS cells grown for 48 h under conditions indicated. Each bar represents the mean of at least three independent experiments with error bars representing the standard deviation *P<0.05; **P<0.01 (Student’s *t*-test). (D) Negative correlation between reduced glutathione levels and ROS levels in NIH3T3 and NIH-RAS cells grown under conditions indicated. Linear regression curves are not parallel with a 99.9% confidence interval; Student’s *t*-test. (E) Crystal violet staining of NIH3T3 and NIH-RAS cells plated at the density of 9000 cells/cm^2^, grown for 72 h under methionine deprivation and then for 48 h in standard medium.

Contrary to the behavior of cells perturbed by cysteine limitation or deprivation, methionine perturbation only weakly enhanced apoptosis in cells, slightly more in NIH-RAS cells (Fig 3A). Methionine limitation and deprivation increased ROS levels, methionine limitation having a significantly stronger effect in NIH-RAS cells (Fig 3B). As a likely consequence, GSH levels under limiting methionine were lower than in standard medium (Fig 3C) and inversely correlated with ROS levels (Fig 3D). By contrast, total glutathione levels (GSH+GSSG) under limiting methionine were similar to those found in standard condition, consistently with the presence in the medium of the glutathione precursor cysteine (Fig 3C). The high cell mortality under methionine deprivation hindered the measurement of glutathione levels. These results demonstrated that changes in ROS and reduced glutathione levels under methionine limitation (and, likely, in methionine deprivation) do not depend on alterations in glutathione biosynthesis. It is noteworthy that supplementation of 4 mM GSH to cells growing in methionine-free medium (-Met+GSH growth condition) resulted in decreased ROS levels in both cell lines (Fig 3B), however, neither NIH3T3 nor NIH-RAS cells were able to grow (Fig 1A, 1C and 1D), and NIH-RAS cells did not form *foci* (Fig 1E).

A GSH *versus* ROS plot (Fig 3D) confirms that GSH and ROS levels are inversely correlated (which is not unexpected) and further shows that limitation of sulfur-containing amino acids results in a more dramatic decrease of GSH as a function of ROS concentration in NIH-RAS compared to NIH3T3 cells.

Supplementation of methionine to cells grown for 72 hours in methionine-free medium resulted in a significant growth recovery, reinforcing the notion that methionine deprivation exerted a cytostatic effect (arrest of cell proliferation) in both NIH3T3 and NIH-RAS cell lines (Fig 3E).

**Fig 4:**
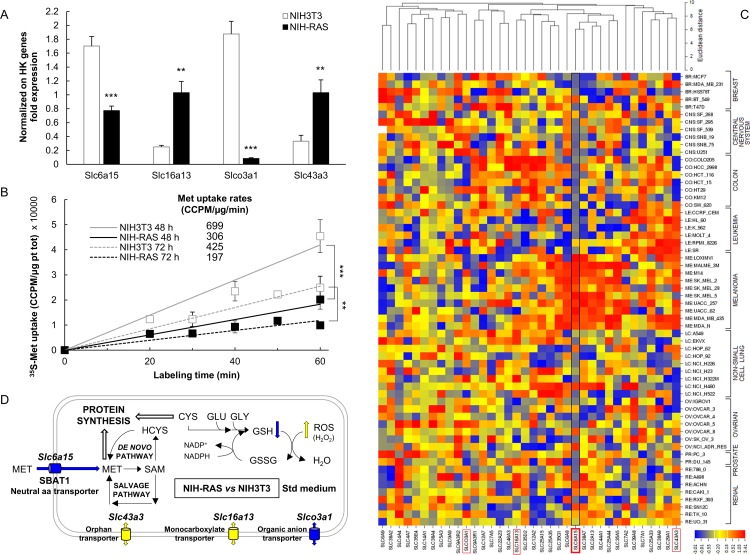
Methionine transport and solute carrier expression in mouse fibroblasts and in NCI-60 panel. (A) Semiquantitative RT-PCR results for NIH3T3 and NIH-RAS cells grown for 48 h in standard medium performed in triplicate on genes showing at least a two-fold change between NIH-RAS *vs* NIH3T3 cells in each of the two Affymetrix independent experiments (S5 Fig). (B) Labeled amino acid (^35^S-methionine) uptake rate in exponential and confluent cells (48 and 72 h of growth in standard medium, respectively), measured after 20’-40’-60’ (for exponential cells) and after 30’-50’-60’ (for confluent cells) of labeling with 0.025 mCi/ml ^35^S-Met. Radioactivity values, expressed as CCPM (corrected counts per minute), were normalized on total protein content and plotted against labeling time. Results are mean +/- standard deviation of three independent experiments. **P<0.01; ***P<0.001 (Student’s *t*-test). (C) The mRNA expression data for the NCI-60 human tumor cell lines were retrieved from CellMiner relational database [59]. These expression data were inputted in CIMminer [60] to generate a heat map, as described in Materials and Methods. Here are highlighted the names of the genes whose expression was statistically different between NIH3T3 and NIH-RAS cells, with a particular emphasis on the data related to *SLC6A15* gene. (D) Concept map of cysteine and methionine metabolism in NIH3T3 and NIH-RAS cells.

We analyzed genome-wide transcriptional profiling datasets for NIH3T3 and NIH-RAS cells (available in NCBI GEO database; accession GSM741354-GSM741361 for NIH3T3 cells and GSM741368-GSM741375 for NIH-RAS cells), previously obtained in our laboratory with an MG_U74Av2 Affymetrix Gene Chip [41] to identify the pattern of expression of genes encoding solute carriers [56]. The expression of four of these genes was significantly altered in Ras-transformed *versus* normal cells (S5 Fig). One of these genes is *Slc6a15* that encodes SBAT1, an amino acid transporter exhibiting strong preference for branched chain amino acids and methionine [57,58]. RT-PCR analysis of the expression of these four genes in normal and transformed fibroblasts (Fig 4A) validated Affymetrix results, clearly indicating that in NIH-RAS cells the expression of *Slc6a15* is down-regulated. Notably, over-expression in NIH-RAS cells of the Ras inhibitor GEF-DN determined a significant increase in the expression of *Slc6a15* (S3C Fig). Consistently with the strong, but not always complete reversion of Ras-dependent phenotypes induced by GEF-DN expression [45], up-regulation of *Slc6a15* expression is strong, but possibly not complete compared to NIH3T3 cells. Our data thus indicate that sensitivity to methionine limitation (S3A and S3B Fig) and expression of the SBAT1-encoding *Slc6a15* gene (S3C Fig) are regulated by the activation state of Ras.

To confirm that methionine transport is impaired in NIH-RAS cells as suggested by transcriptional analysis, we assayed methionine uptake in NIH3T3 and NIH-RAS cells by using a ^35^S-methionine incorporation assay. NIH-RAS cells showed a significantly reduced incorporation of ^35^S-methionine per unit of protein in both exponential and confluent growth conditions (Fig 4B). The combined transcriptional and biochemical analyses therefore suggest that down-regulation of *Slc6a15* gene expression and ensuing decreased methionine transport activity in Ras-transformed cells could be the reason for their higher sensitivity to methionine limitation.

## Discussion

Cancer cells show metabolic dependencies that distinguish them from their normal counterparts [14]. Personalized targeting of cancer metabolism that accounts for differences in genetic, epigenetic and environmental factors (i.e., nutrient availability) may lead to major advances in tumor therapy [61]. In this paper we perform nutrient perturbation of the supply of the proteinogenic sulfur-containing amino acids methionine (a potential cysteine precursor) and cysteine (a GSH precursor) of normal, Ras-transformed and reverted mouse fibroblasts to highlight any differential biological response due to the activation state of Ras oncoprotein.

We show that cysteine deprivation causes cell proliferation arrest in both normal and Ras-transformed mouse fibroblasts even in presence of methionine in the culture media. Although databases of metabolic pathway maps, like KEGG ([62,63]), Human Metabolic Atlas ([64]), Reactome ([65]) or Recon2 ([66]) annotate methionine-to-cysteine conversion for all considered cell types, we show that the biosynthetic pathway of cysteine from methionine is not active in mouse fibroblasts (Fig 4D and S1 Fig). In fact, the methionine-to-cysteine pathway may be active only in cells from splanchnic organs, described as important sites of trans-methylation and trans-sulfuration of dietary methionine for cysteine synthesis [36]. These data are in keeping with previous results demonstrating the dependency from cystine for growth of several human diploid cell lines (human fibroblasts), not able to utilize cystathionine *in lieu* of cystine, likely as a consequence of deficient cystathionase activity [67].

Cysteine deprivation is accompanied by an increase in ROS levels, which could be due to an enhancement of mitochondrial metabolism, and particularly of oxidative phosphorylation-associated proton leakage, induced by energetic stress and increased ATP-demand. This redox unbalance induced by nutritional stress has a pivotal role in up-regulating cellular repair processes and other protective systems (e.g., chaperones) and in driving autophagy, a major mechanism by which starving cells mobilize and reallocate intracellular nutrient resources in order to maintain processes necessary for survival during growth-unfavorable conditions [68].

Cysteine deprivation causes apoptotic cell death. Apoptosis induced by cysteine-withdrawal is essentially due to increased oxidative stress caused by glutathione deprivation. Non-cysteine containing anti-oxidants effectively rescue oxidative stress, but cannot rescue cell death induced by cysteine deprivation. Supplementing reduced glutathione to cysteine-deprived cells not only restores redox homeostasis (and suppresses apoptosis), but also partially restores cell growth, indicating that in mouse fibroblasts GSH can be used as a cysteine reservoir to maintain protein synthesis under nutritional stress. However, high concentrations of GSH have a toxic effect, stressing the notion that to maximize viability a proper balance between ROS and antioxidants needs to be obtained [54]. Under standard cysteine conditions, severe inhibition of glutathione biosynthesis increases oxidative stress, but has moderate effects on viability. Growth defects induced by cysteine limitation are synergistically increased by inhibiting glutathione synthesis, the more so in NIH-RAS cells, indicating that the growth defects induced by cysteine limitation are largely–though not exclusively–due to cysteine utilization in the synthesis of glutathione. The differential sensitivity of NIH3T3 and NIH-RAS cells to both protective and toxic effects of glutathione may depend on the higher glutathione content of NIH3T3 cells.

The role of cysteine in cancer is controversial. While some authors report that human tumor growth is associated with decreased plasma levels of cysteine and homocysteine [69], more recently other authors demonstrated that antioxidants such as N-acetylcysteine (a direct precursor of cysteine) can accelerate tumor progression by decreasing ROS levels, DNA damage and p53 (a tumor suppressor gene) levels in cancer [70].

The increase in ROS levels under methionine deprivation in both NIH3T3 and NIH-RAS cell lines is not followed by a significant increase in neither apoptosis nor necrosis. While cell growth of normal and Ras-transformed cells was similarly compromised by methionine deprivation, methionine limitation mostly affected NIH-RAS cells.

Some cancers show methionine dependence, a feature firstly noted in xenograft rodents in response to a methionine-free diet [71]. Since then normal cells have been reported to be more resistant to external methionine limitation [34,72]. Methionine dependence might be correlated with inability of methionine-restricted cells to cope with demand for SAM, a major methionine product [34]. This “SAM-checkpoint” may protect cellular integrity and maintain epigenetic stability, since it stops cell cycle progression when intracellular SAM concentrations are insufficient to sustain the methylation reactions necessary for normal cell physiology [34]. Several drugs that target the enzymes that are involved in the post-translational modifications of histones and DNA, cell survival, proliferation and stem cell function [33,73,74] are being evaluated pre-clinically or in early-stage clinical trials [75].

Both a deficiency and an excess of the dietary levels of methionine can result in either genomic instability, which leads to diseases such as cancer, or changes in gene expression, which lead to alterations in metabolism [76], including improvement of hepatic lipid and glucose metabolism and induction of adiposity resistance [76]. Some cancer cells show a high activity of the methionine cycle that promotes chemo-resistance and evasion from apoptosis [77], whereas normal cells are relatively resistant to dietary methionine restriction: therapies to block the methionine cycle in transformed cells may thus represent a safe and effective strategy to fight cancer [39,77]. Dietary methionine restriction, used alone or in combination with other treatments, impaired cancer growth and carcinogenesis in human patients [78,79] or in rodents [80,81,82]. However, one *caveat* is that methionine restriction must be closely regulated, because methionine is an essential amino acid and a long use of diets extremely poor in methionine could be extremely toxic and cause death. Dietary methionine restriction (achievable in humans with a predominantly vegan diet) may have an additive healthy effect if combined with calorie restriction, by limiting glucose [82]. The potential of methionine depletion in enhancing the anti-cancer effect of chemotherapeutic agents on drug-resistant tumors and cell lines has also been reported [83].

Sensitivity to methionine limitation of mouse fibroblasts and expression of the SBAT1-encoding *Slc6a15* gene are regulated by the activation state of Ras (Fig 4A and S3 Fig), resulting in decreased methionine uptake in NIH-RAS (Fig 4B). Remarkably, expression of the ortholog human gene—*SLC6A15—*is mostly down-regulated in the NCI-60 cells panel, the US National Cancer Institute (NCI) panel of 60 human cancer cell lines grown in culture [84] (Fig 4C). An exception is represented by melanoma cells, in which *SLC6A15* is highly up-regulated. Therefore, the use of methionine uptake as a marker for proliferative activity in substitution of fluoro-deoxyglucose [85,86], or therapeutic use of dietary methionine restriction would benefit from knowledge of the expression of methionine transporters.

*Slc6a15* and its human ortholog belong to a large family (over 450 members) of solute carrier proteins (SLCs) controlling import/export of nutrients, cofactors, ions and many drugs. While many SLCs have not yet well characterized, a quarter of their encoding genes has been associated with human diseases and 26 different SLCs are the targets of known drugs, or drugs in development [87,88]. An increase in amino acid transport may be expected in cancer, most likely as the result of increased amino acid demand for energy, protein synthesis and cell division: surprisingly, S5 Fig shows that SLC-encoding genes down-regulated in NIH-RAS compared to NIH3T3 cells are enriched in genes encoding amino acid transport, particularly of neutral amino acids (e.g. the SBAT1-encoding *Slc6a15* gene).

In conclusion, we show that limitation of sulfur-containing amino acids results in a more dramatic perturbation of the oxidoreductive balance in *K-ras*-transformed cells compared to NIH3T3 cells (Fig 3D). Growth defects induced by cysteine limitation in mouse fibroblasts are largely–though not exclusively–due to cysteine utilization in the synthesis of glutathione, mouse fibroblasts requiring an exogenous cysteine source for protein synthesis. We show for the first time a correlation between Ras-transformation and defects in methionine transport that affect the dependence of *K-ras*-transformed mouse fibroblasts for this amino acid. Therapeutic regimens of cancer involving modulation of methionine metabolism could be more effective in cells with limited methionine transport capability. To further understand nutrient interactions (such as methionine and glucose restriction), to study the correlation between methionine metabolism and cell signaling and to design a precision medicine approach taking into account the specific nutritional dependencies of a patient’s cancer, we consider essential to unravel the underlying networks by using an integrated, Systems Biology approach.

## Materials and Methods

### Cell culture

Three cells lines have been used in this paper, namely normal NIH3T3 mouse fibroblasts (obtained from the ATCC, Manassas, VA, USA), a K-Ras-transformed normal-derived cell line -that we refer to as NIH-RAS [44,89]- and NIH-RAS cells stably transfected with a pcDNA3-based vector expressing a dominant negative mutant of the Ras-specific guanine nucleotide exchange factor RasGRF1 (RasGRF1W1056E, here simply named GEF-DN) with Ras-sequestering property [44,45,90]. These cell lines proved to be a valid cellular model for studying Ras-dependent transcriptional reprogramming [41], and metabolic rewiring [23,42,43]. Both control and *ras*-transformed NIH3T3 have been passaged a similar number of times, taking care to refreeze the cell lines immediately and to use them for a limited number of passages. The cell lines are periodically assayed to check that the major properties of the cells do not change over time, that the major transformation-related phenotypes are retained and *ras*-dependent (see S2 Fig and accompanying text). The cell lines were routinely grown in Dulbecco’s modified Eagle’s medium (Invitrogen Inc., Carlsbad, CA, USA) containing 10% newborn calf serum, 4 mM glutamine, 100 U/ml penicillin and 100 mg/ml streptomycin (standard medium), at 37°C in a humidified atmosphere of 5% CO_2_. Cells were passaged using trypsin-ethylenediaminetetraacetic acid (EDTA) (Invitrogen Inc., Carlsbad, CA, USA) and maintained in culture before experimental manipulation.

### Cell proliferation analysis

Cells were plated at the density of 3000 cells/cm^2^ in standard medium and incubated overnight at 37°C and 5% CO_2_. After 18 h, cells were washed twice with phosphate-buffered saline (PBS) and, to verify the response to the cysteine or methionine deprivation, cells were incubated in medium without cysteine and methionine (Invitrogen Inc., Carlsbad, CA, USA), possibly supplemented with limiting concentration of cysteine (0.025, 0.05, 0.1 mM) or methionine (0.025, 0.1 mM) (Sigma Aldrich Inc.) or with antioxidants glutathione (0.08, 0.2, 0.8, 2, 4, 16 mM) or MitoTEMPO (10 μM) (Sigma Aldrich Inc.). To measure cell proliferation, cells were treated with trypsin at 0, 3, 6, 24, 30, 48, 54, 72 hours after medium change. Viable (i.e., unstained) cells were counted in a Bürker chamber after staining with 0.5% trypan blue. In amino acid re-feeding and *foci* formation experiments, qualitative evaluation of cell proliferation was obtained by staining with 0.2% Crystal violet (diluted in water from Giemsa Stain 0.4%, Sigma Aldrich Inc.). After 45 minutes of incubation in the dark at RT, cells were washed twice with water, photographed, and counted.

### *Foci* formation assay

Cells were plated at the density of 30 cells/cm^2^ in standard medium and incubated overnight at 37°C and 5% CO_2_. After 18 h, cells were washed twice with phosphate-buffered saline (PBS) and, to test the ability of forming *foci* of NIH3T3 and NIH-RAS under nutritional modulation, cells were incubated for 9 days in medium without cysteine and methionine (Invitrogen Inc., Carlsbad, CA, USA), possibly supplemented with limiting concentration of cysteine (0.025, 0.05 mM) or methionine (0.025, 0.1 mM) (Sigma Aldrich Inc.) or with 4 mM reduced glutathione (Sigma Aldrich Inc.). After 9 days, cells were washed with PBS and fixed with paraformaldehyde 4%, then washed with ice-cold PBS and stained with 0.2%. Crystal violet, photographed as described above and the number of *foci* counted.

### Determination of intracellular ROS

Intracellular accumulation of H_2_O_2_ and O2^•-^ was determined after 48 h from medium change with 2’,7’-dichlorodihydrofluoresceine diacetate (Sigma Aldrich Inc.). The cells were incubated for 30 minutes at 37°C with H_2_DCFDA 10 mM, treated with trypsin, resuspended in PBS supplemented with NCS 10% (Invitrogen Inc., Carlsbad, CA, USA) and acquired by FACScan (Becton-Dickinson), using the Cell Quest software (BD Bioscience). The percentage of ROS-producing cells was calculated for each sample and corrected for autofluorescence obtained from samples of unlabeled cells.

### Apoptosis Assay

Cells were plated at the density of 3000 cells/cm^2^ in standard medium and incubated overnight at 37°C and 5% CO_2_. After 18 h, cells were washed twice with phosphate-buffered saline (PBS) and incubated for 30 hours in medium without cysteine and methionine (Invitrogen Inc., Carlsbad, CA, USA), possibly supplemented with limiting concentrations of cysteine (0.025, 0.05 mM) or methionine (0.025 and 0.1 mM) (Sigma Aldrich Inc.) or with antioxidants glutathione (4 mM) or MitoTEMPO (10 μM) (Sigma Aldrich Inc.). For apoptosis analysis, 1 × 10^6^ cells (adherent and in suspension cells) were collected, stained with Annexin V-FITC (Immunotools, GmbH) and propidium iodide (Sigma Aldrich Inc.) and analyzed by FACScan (Becton-Dickinson) using the FL1 and FL2 channels. Data analysis was performed with Flowing Software.

### Determination of glutathione levels

For reduced and total glutathione measurements, cells were plated at the density of 3000 cells/cm^2^ in standard medium and incubated overnight at 37°C and 5% CO_2_. After 18 h, cells were washed twice with phosphate-buffered saline (PBS) and incubated for 48 h in standard medium or under limitation of cysteine or methionine. Cells were then treated with trypsin, collected, washed twice with PBS and lysed through freeze-and-thaw cycles. Samples were deproteinized with a 5% 5-sulfosalicylic acid solution, centrifuged to remove the precipitated protein and assayed for glutathione. GSH measurement was an optimization of Tietze’s enzymatic recycling method [55], in which GSH is oxidized by the sulfhydryl reagent 5,5’-dithio-bis(2-nitrobenzoic acid) (DTNB) to form the yellow derivative 5’-thio-2-nitrobenzoic acid (TNB), measurable at 412 nm and the glutathione disulfide (GSSG) formed is recycled to GSH by glutathione reductase in the presence of NADPH. The amount of glutathione in the samples was determined through a standard curve of reduced glutathione. Glutathione levels were normalized to protein content measured by Bradford assay (Bio-Rad reagent) on an aliquot of cell extract collected before deiproteinization.

### Methionine transport assays

NIH3T3 and NIH-RAS cells were seeded at the density of 3000 cells/cm^2^ and incubated overnight at 37°C and 5% CO_2_, then medium change was done after 18 h. At 48 h (exponential growth condition) and 72 h (confluent growth condition), standard medium was replaced with 0.4 ml labeling medium (cysteine and methionine-free medium + 0.025 mCi/ml ^35^S-Met, PerkinElmer), that was removed after 20-40-60 minutes or 30-50-60 minutes at 37°C and 5% CO_2_. Cells were then washed once with cold PBS and scraped after adding lysis buffer. Cell lysates were centrifuged and an aliquot spotted on Whatman Glass Microfiber filters (Sigma Aldrich Inc.). To the remaining volume, 1 volume of cold 20% TCA (Sigma Aldrich Inc.) was added and, after 30 minutes in ice, samples were spotted on filters and washed twice with cold 10% TCA and ethanol (Sigma Aldrich Inc.). Air-dried filters were transferred to vials containing Ultima Gold MV scintillation fluid (PerkinElmer) and radioactivity measured in a beta-counter (Wallac Microbeta Trilux, PerkinElmer). Averages of technical triplicates for cell lysates (representing amino acid uptake) were calculated and the resulting values were normalized on total protein content, measured by using QuantiPro^TM^ BCA Assay Kit (Sigma Aldrich Inc.).

### RNA extraction and semi quantitative RT-PCR analysis

Cells were plated at the density of 3000 cells/cm^2^ in standard medium and incubated overnight at 37°C and 5% CO_2_. After 18 h, cells were washed twice with phosphate-buffered saline (PBS) and incubated for 48 h in standard medium. RNA was then extracted from cells by using the Quick-RNA™ MicroPrep kit (Zymo Research). Total RNA was reverse-transcribed with oligo-dT by using the iScript cDNA Synthesis Kit (Bio-Rad Laboratories). The RT product (0.5 μg) was amplified with primer pairs specific for the genes studied. As internal control of PCR assays, specific primers for 18S and β-actin transcripts were used. Primers used: *Slc6a15* forward: 5’-GCATCGGAAGAATTTCTGAGC-3’, reverse: 5’-AGCGACGAATGATGAACACC-3’; *Slco3a1* forward: 5’-GAGTTAGCCTATCCTTGTTG-3’, reverse: 5’-GACAGAACATCACCTTACAA-3’; *Slc16a13* forward: 5’-ACCTGAGTATTGGGCTGCTG-3’, reverse: 5’-CCATGGTCGGAGTGAAGGT-3’; *Slc43a3* forward: 5’-CACCTTGTTGACTGGACTCTTG-3’, reverse: 5’-CCAGGGTAAAGATGAGTGAGAAC-3’.

### Generation of the heat map of solute carrier gene expression profiles in NCI-60 cell lines

The heat map, or Clustered Image Map (CIM), was generated with CIMminer by selecting the one matrix option. The rows of the matrix were the different cell lines and the columns (each representing a solute carrier gene) were clustered according to Average Linkage algorithm and to Euclidean distance measure. Data values were mapped to colors using the quantile method: the weight range of data values was divided into intervals each containing approximately the same number of data points, thus effectively spreading out the color differences between data values that were present in regions with a large number of values.

## Supporting Information

S1 Fig Methionine and cysteine metabolism in mouse fibroblasts. Methionine is partitioned between protein synthesis, *de novo* and recycling pathway, where it is converted to S-adenosylmethionine (SAM). SAM is converted to S-adenosylhomocysteine (SAH) during methylation of DNA and a large range of proteins and other molecules. SAH is then hydrolyzed to homocysteine (Hcy) in a reversible reaction. Under normal conditions, approximately 50% of Hcy is re-methylated to form methionine that, in most tissues, occurs via methionine synthase. In the trans-sulfuration pathway, Hcy is metabolized to form cystathionine, which is the immediate precursor to cysteine. Besides from methionine, cysteine can be synthesized from serine. The sulfur is derived from methionine, which is converted to homocysteine through the intermediate SAM. Cystathionine beta-synthase then combines homocysteine and serine to form the asymmetrical thioether cystathionine. The enzyme cystathionine gamma-lyase converts the cystathionine into cysteine and alpha-ketobutyrate. The trans-sulfuration pathway is not active in all cells, and in human is active essentially only in cells from splanchnic organs. Here we demonstrated that mouse embryonic fibroblasts are not able to convert methionine into cysteine. For this reason the trans-sulfuration reaction is highlighted in grey.
(PDF)

S2 Fig Ras and MAPK activation state and expression levels in cellular models used in the paper: NIH3T3, NIH-RAS, NIH-RAS pGEF-DN and NIH-RAS pcDNA3. Expression levels of Total Ras proteins (A) and MAPKs p42 and p44 (B) in cell lysates of pull down assay. Antibodies directed against Ras (sc259 Santa Cruz), Phospho-p44/42 MAPK (Erk1/2) (Thr202/Tyr204) (Cell Signaling #9101) and p44/42 MAPK (Erk1/2) (Cell Signaling #9102) were used. (C) Ras–GTP eluted from GST–RBD–glutathione–sepharose, pre-incubated with cell lysates. Pull down assay was performed as described in [7]. (D) Quantification of the Ras–GTP amount after normalization over total Ras. Data are normalized over the Ras-GTP/total Ras ratio in NIH3T3 taken equal to 100. Data shown are mean +/- standard deviation of two independent experiments. (E) Morphological analysis of the different cell lines. (F) Phospho-p44/42 MAPK level in cell lysates, determined by ELISA assay performed using PathScan® Phospho-p44/42 MAPK (Thr202/Tyr204) (Cell Signaling). Data shown are mean +/- standard deviation of two independent experiments. (F) 100X magnification of a *focus* generated by NIH-RAS cells in *foci* formation assay shown in Fig 1.
(PDF)

S3 Fig Over-expression of GEF-DN reverts sensitivity to methionine limitation in NIH-RAS cells and partially rescues the defect in the expression of *Slc6a15* gene encoding methionine transporter SBAT1. (A) Cell proliferation of NIH3T3, NIH-RAS, NIH-RAS pGEF-DN and NIH-RAS pcDNA3 cells grown in media with different concentrations of methionine and counted daily for 72 h of growth under conditions indicated. Plotted data are mean +/- standard deviation. computed from three independent experiments. (B) Relative to t = 0 cell proliferation of NIH3T3, NIH-RAS, NIH-RAS pGEF-DN and NIH-RAS pcDNA3 cells grown for 72 h in media with different concentrations of methionine, as indicated in (A). Part of the data in (B) are present in Fig 1D. (C) Semi-quantitative RT-PCR results for NIH3T3, NIH-RAS, NIH-RAS pGEF-DN and NIH-RAS pcDNA3 cells grown for 48 h in standard medium performed in triplicate on genes showing at least a two-fold change between NIH-RAS *vs*. NIH3T3 cells in each of the two Affymetrix independent experiments (S5 Fig). *P<0.05; **P<0.01; ***P<0.001 (Student’s *t*-test).
(PDF)

S4 Fig Cell proliferation and qualitative ROS levels under different methionine concentrations and in cysteine-limiting or -depleted medium (possibly supplemented with antioxidants glutathione and MitoTEMPO or with GSH synthesis inhibitor BSO). For all the experiments, MitoTEMPO and buthionine sulfoximine (BSO) were used at the concentration of 10 μM and 100 μM. (A-B) Cell proliferation of NIH3T3 and NIH-RAS cells grown in media supplemented with different concentrations of methionine and cysteine with or without antioxidants glutathione or MitoTEMPO and counted after 72 h (A) and 30 h (B) of growth under conditions indicated. Part of the data in (A) are present in Fig 1D. Plotted data are mean +/- standard deviation computed from three independent experiments. *P<0.05 (Student’s *t*-test). (C) Cell proliferation of NIH3T3 and NIH-RAS cells under conditions indicated. (D) Qualitative evaluation of ROS levels in NIH3T3 and NIH-RAS cells upon staining with DCFDA and analysis with a fluorescence microscope.
(PDF)

S5 Fig Solute carriers differentially expressed between NIH3T3 and NIH-RAS cells. Genome-wide transcriptional profiling datasets for NIH3T3 and NIH-RAS cells (available in NCBI GEO database; accession GSM741354-GSM741361 for NIH3T3 cells and GSM741368-GSM741375 for NIH-RAS cells), previously obtained in our laboratory with an MG_U74Av2 Affymetrix Gene Chip [41], were filtered for all genes encoding for solute carriers. Then, to identify genes whose expression was significantly altered in Ras-transformed *versus* normal cells (here represented in bold), a two-fold and a <0.05 cut-offs on Fold Changes and on *p*-values were used, respectively. In this Figure are represented all transporter genes with a fold change ≥2 (about 20% of all transporter genes) irrespective of their *p*-values. Gene Ontology (GO) enrichment based on molecular function was performed with GoTermFinder (http://go.princeton.edu/cgi-bin/GOTermFinder) and genes encoding for amino acid transporters were colored in magenta, while genes encoding for ion transporters were colored in grey.
(PDF)

S1 Table Mass duplication times under different nutritional perturbations. Mass duplication times (MDT) for NIH3T3 and NIH-RAS under different methionine or cysteine concentrations (possibly supplemented with GSH) were calculated on semi-logarithmic curves represented in Fig 1A and 1B. Then, Student’s *t*-test was performed on linear regression curves for each nutritional condition that allowed cell growth. A = not parallel to linear regression curve of NIH-RAS cells in standard medium (99% CI); B = not parallel to linear regression curve of NIH3T3 cells in standard medium (99.9% CI); C = not parallel to linear regression curve of NIH-RAS cells in standard medium (99.9% CI); D = not parallel to linear regression curve of NIH3T3 cells in standard medium (99% CI); E = not parallel to linear regression curve of NIH3T3 cells in standard medium (99.9% CI). CI = confidence interval.
(PDF)
