# Supplementary figures and images for: K-Ras Activation Induces Differential Sensitivity to Sulfur Amino Acid Limitation and Deprivation and to Oxidative and Anti-Oxidative Stress in Mouse Fibroblasts
Source: PLoS One. 2016 Sep 29;11(9):e0163790. doi: 10.1371/journal.pone.0163790 (PMC5042513; doi:10.1371/journal.pone.0163790)

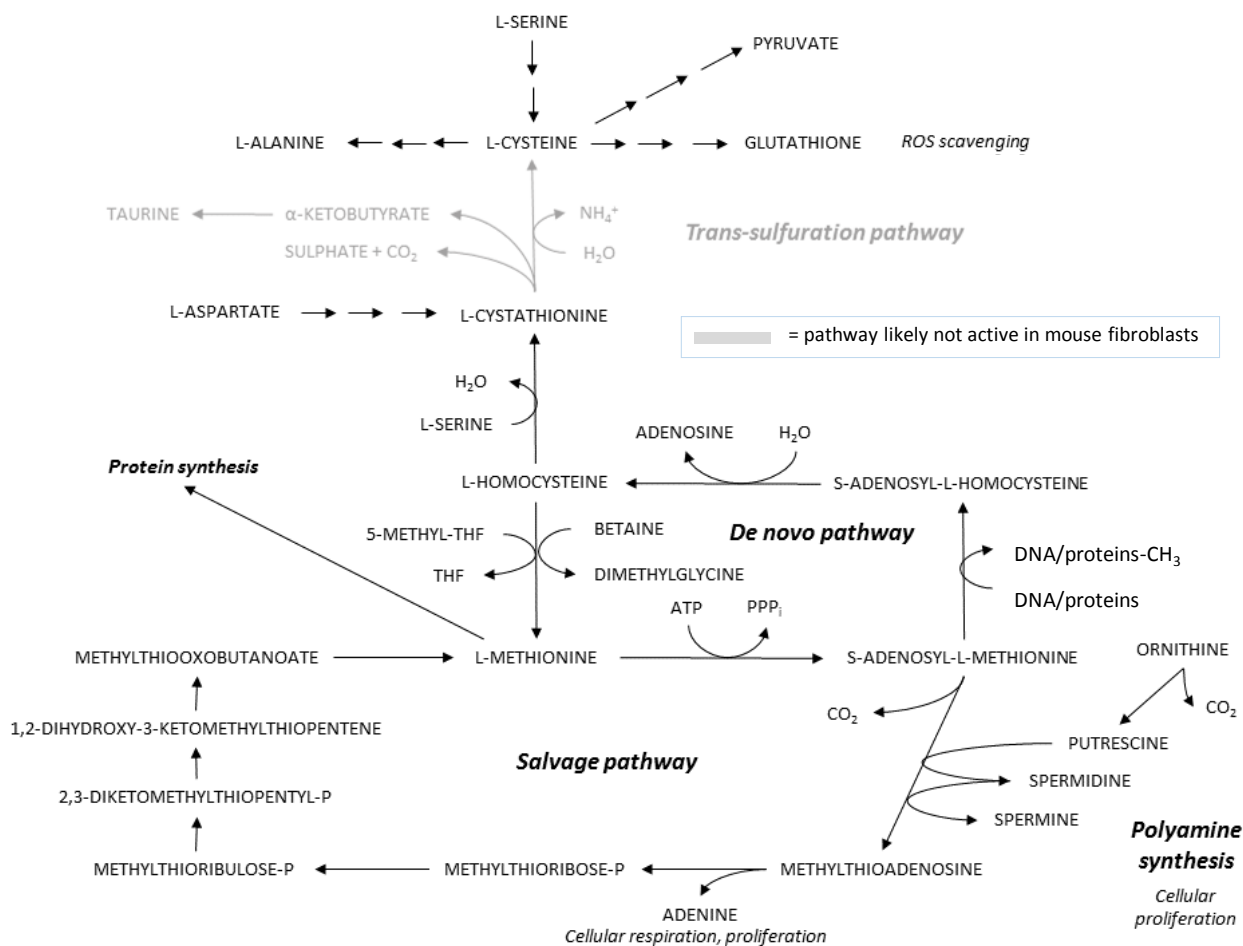

**S1 Fig. Methionine and cysteine metabolism in mouse fibroblasts.**

Supplement: S1 Fig — Methionine is partitioned between protein synthesis, de novo and recycling pathway, where it is converted to S-adenosylmethionine (SAM). SAM is converted to S-adenosylhomocysteine (SAH) during methylation of DNA and a large range of proteins and other molecules. SAH is then hydrolyzed to homocysteine (Hcy) in a reversible reaction. Under normal conditions, approximately 50% of Hcy is re-methylated to form methionine that, in most tissues, occurs via methionine synthase. In the trans-sulfuration pathway, Hcy is metabolized to form cystathionine, which is the immediate precursor to cysteine. Besides from methionine, cysteine can be synthesized from serine. The sulfur is derived from methionine, which is converted to homocysteine through the intermediate SAM. Cystathionine beta-synthase then combines homocysteine and serine to form the asymmetrical thioether cystathionine. The enzyme cystathionine gamma-lyase converts the cystathionine into cysteine and alpha-ketobutyrate. The trans-sulfuration pathway is not active in all cells, and in human is active essentially only in cells from splanchnic organs. Here we demonstrated that mouse embryonic fibroblasts are not able to convert methionine into cysteine. For this reason the trans-sulfuration reaction is highlighted in grey. (PDF) [file pone.0163790.s001.pdf]

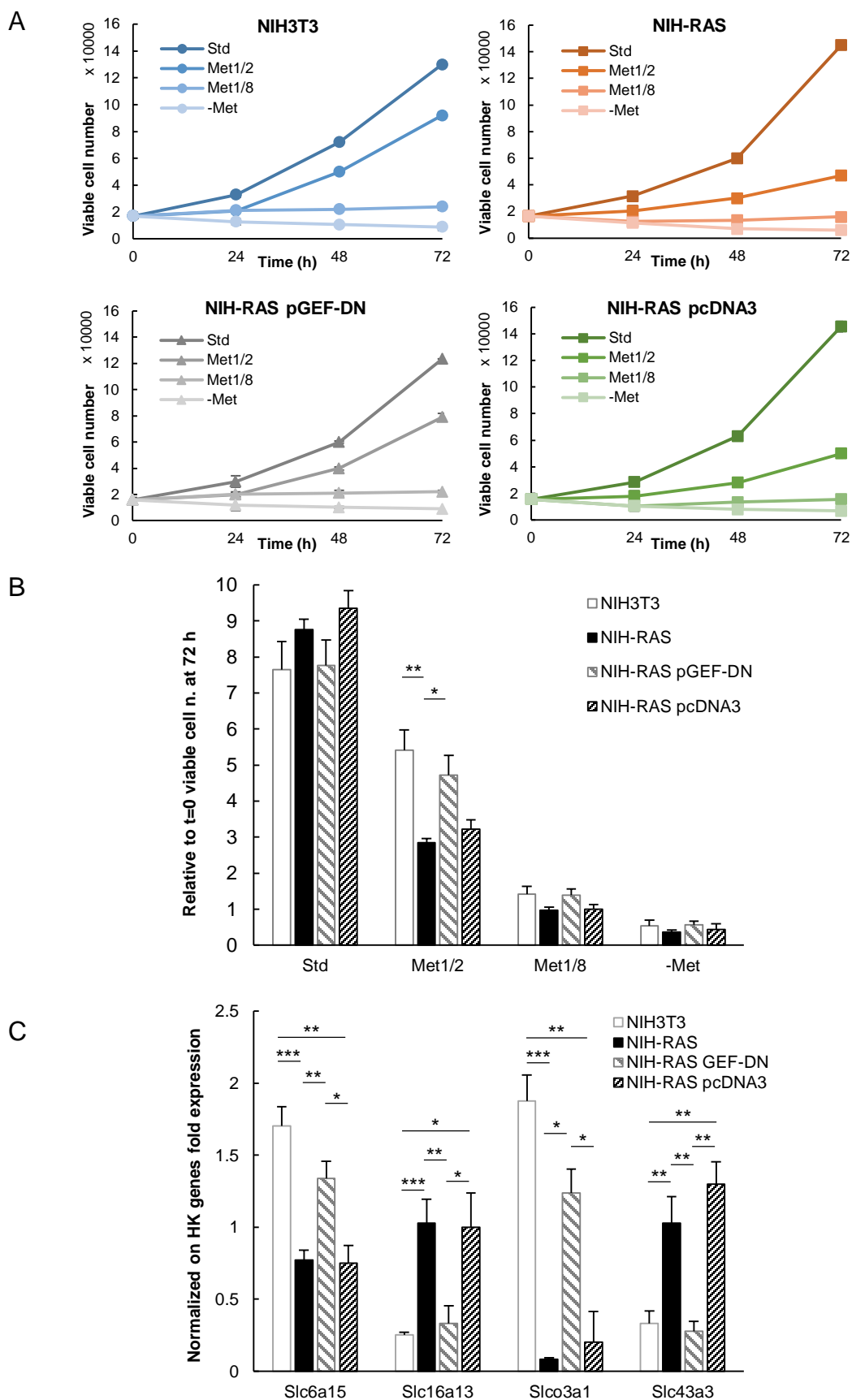

Supplement: S3 Fig — (A) Cell proliferation of NIH3T3, NIH-RAS, NIH-RAS pGEF-DN and NIH-RAS pcDNA3 cells grown in media with different concentrations of methionine and counted daily for 72 h of growth under conditions indicated. Plotted data are mean +/- standard deviation. computed from three independent experiments. (B) Relative to t = 0 cell proliferation of NIH3T3, NIH-RAS, NIH-RAS pGEF-DN and NIH-RAS pcDNA3 cells grown for 72 h in media with different concentrations of methionine, as indicated in (A). Part of the data in (B) are present in Fig 1D. (C) Semi-quantitative RT-PCR results for NIH3T3, NIH-RAS, NIH-RAS pGEF-DN and NIH-RAS pcDNA3 cells grown for 48 h in standard medium performed in triplicate on genes showing at least a two-fold change between NIH-RAS vs. NIH3T3 cells in each of the two Affymetrix independent experiments (S5 Fig). *P<0.05; **P<0.01; ***P<0.001 (Student’s t-test). (PDF) [file pone.0163790.s003.pdf]
