# Supplementary material for: K-Ras Activation Induces Differential Sensitivity to Sulfur Amino Acid Limitation and Deprivation and to Oxidative and Anti-Oxidative Stress in Mouse Fibroblasts
Source: PLoS One. 2016 Sep 29;11(9):e0163790. doi: 10.1371/journal.pone.0163790 (PMC5042513; doi:10.1371/journal.pone.0163790)
Supplement: S2 Fig — Expression levels of Total Ras proteins (A) and MAPKs p42 and p44 (B) in cell lysates of pull down assay. Antibodies directed against Ras (sc259 Santa Cruz), Phospho-p44/42 MAPK (Erk1/2) (Thr202/Tyr204) (Cell Signaling #9101) and p44/42 MAPK (Erk1/2) (Cell Signaling #9102) were used. (C) Ras–GTP eluted from GST–RBD–glutathione–sepharose, pre-incubated with cell lysates. Pull down assay was performed as described in [7]. (D) Quantification of the Ras–GTP amount after normalization over total Ras. Data are normalized over the Ras-GTP/total Ras ratio in NIH3T3 taken equal to 100. Data shown are mean +/- standard deviation of two independent experiments. (E) Morphological analysis of the different cell lines. (F) Phospho-p44/42 MAPK level in cell lysates, determined by ELISA assay performed using PathScan® Phospho-p44/42 MAPK (Thr202/Tyr204) (Cell Signaling). Data shown are mean +/- standard deviation of two independent experiments. (F) 100X magnification of a focus generated by NIH-RAS cells in foci formation assay shown in Fig 1. (PDF) [file pone.0163790.s002.pdf]

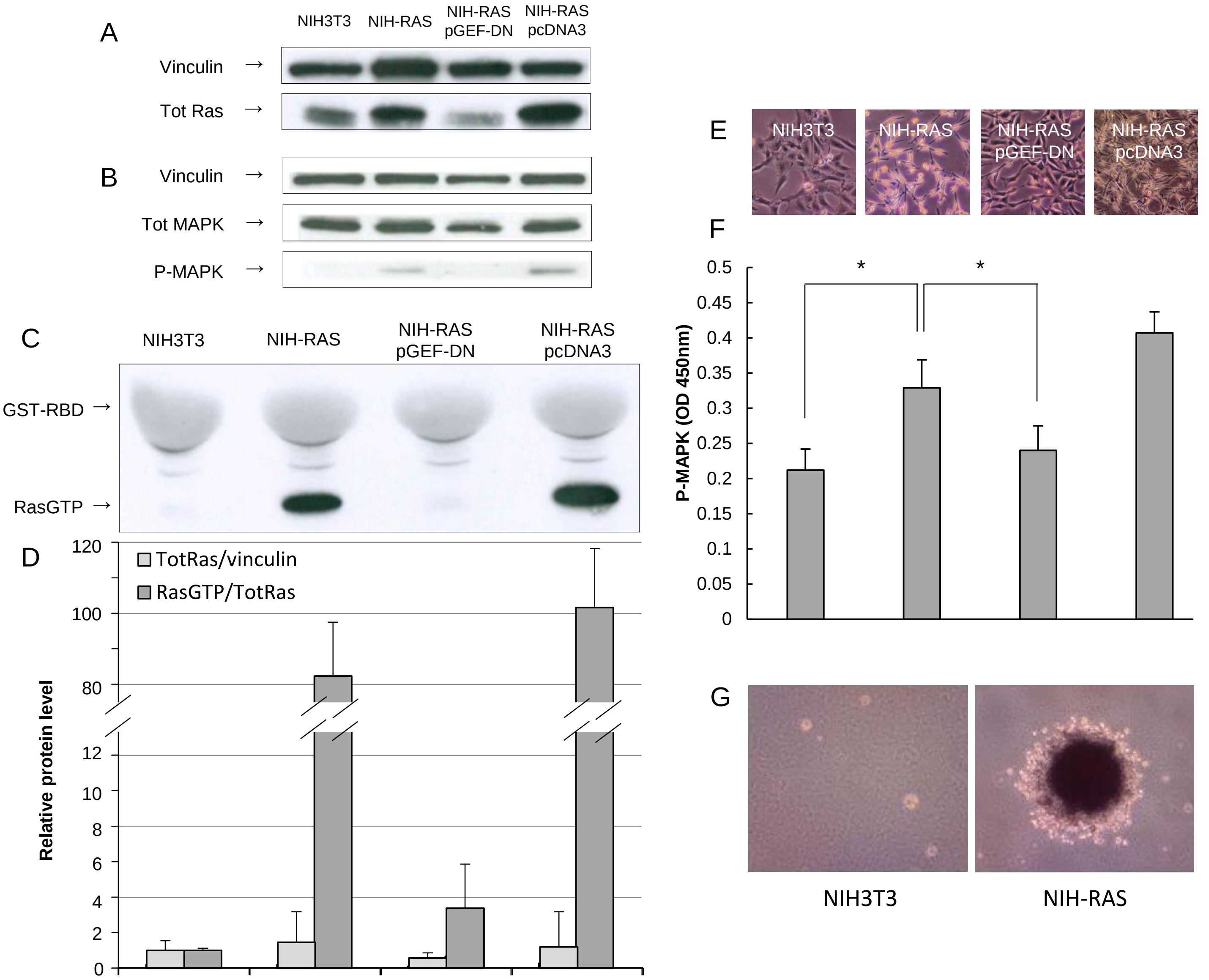

References

Balestrieri et al. (2012). Integrative transcriptional analysis between human and mouse cancer cells provides a common set of transformation associated genes. Biotechnol Adv *30*, 16-29.

Baracca et al. (2010). Mitochondrial Complex I decrease is responsible for bioenergetic dysfunction in K-ras transformed cells. Biochim Biophys Acta *1797*, 314-323.

Bossu et al. (2000). A dominant negative RAS-specific guanine nucleotide exchange factor reverses neoplastic phenotype in K-ras transformed mouse fibroblasts. Oncogene *19*, 2147-2154.

Chiaradonna et al. (2006a). Expression of transforming K-Ras oncogene affects mitochondrial function and morphology in mouse fibroblasts. Biochim Biophys Acta *1757*, 1338-1356.

Chiaradonna et al. (2005). Acquired glucose sensitivity of k-ras transformed fibroblasts. Biochem Soc Trans *33*, 297-299.

Chiaradonna et al. (2006b). Ras-dependent carbon metabolism and transformation in mouse fibroblasts. Oncogene *25*, 5391-5404.

Gaglio et al. (2011). Oncogenic K-Ras decouples glucose and glutamine metabolism to support cancer cell growth. Mol Syst Biol *7*, 523.

Gaglio et al. (2009). Glutamine deprivation induces abortive s-phase rescued by deoxyribonucleotides in k-ras transformed fibroblasts. PLoS One *4*, e4715.

Gaglio et al. (2016). Divergent in vitro/in vivo responses to drug treatments of highly aggressive NIH-Ras cancer cells: a PET imaging and metabolomics-mass-spectrometry study. Oncotarget.

Palorini et al. (2013a). Glucose starvation induces cell death in K-ras-transformed cells by interfering with the hexosamine biosynthesis pathway and activating the unfolded protein response. Cell Death Dis *4*, e732.

Palorini et al. (2013b). Oncogenic K-ras expression is associated with derangement of the cAMP/PKA pathway and forskolin-reversible alterations of mitochondrial dynamics and respiration. Oncogene *32*, 352-362.

Palorini et al. (2013c). Mitochondrial complex I inhibitors and forced oxidative phosphorylation synergize in inducing cancer cell death. Int J Cell Biol *2013*, 243876.

Palorini et al. (2016). Protein Kinase A Activation Promotes Cancer Cell Resistance to Glucose Starvation and Anoikis. PLoS Genet *12*, e1005931.

Pulciani et al. (1985). ras gene Amplification and malignant transformation. Mol Cell Biol *5*, 2836-2841.

Sacco et al. (2012). Novel RasGRF1-derived Tat-fused peptides inhibiting Ras-dependent proliferation and migration in mouse and human cancer cells. Biotechnol Adv *30*, 233-243.

S2 Fig. Ras and MAPK activation state and expression levels in cellular models used in the paper: NIH3T3, NIH-RAS, NIH-RAS pGEF-DN and NIH-RAS pcDNA3.
