# Supplementary material for: K-Ras Activation Induces Differential Sensitivity to Sulfur Amino Acid Limitation and Deprivation and to Oxidative and Anti-Oxidative Stress in Mouse Fibroblasts
Source: PLoS One. 2016 Sep 29;11(9):e0163790. doi: 10.1371/journal.pone.0163790 (PMC5042513; doi:10.1371/journal.pone.0163790)
Supplement: S4 Fig — For all the experiments, MitoTEMPO and buthionine sulfoximine (BSO) were used at the concentration of 10 μM and 100 μM. (A-B) Cell proliferation of NIH3T3 and NIH-RAS cells grown in media supplemented with different concentrations of methionine and cysteine with or without antioxidants glutathione or MitoTEMPO and counted after 72 h (A) and 30 h (B) of growth under conditions indicated. Part of the data in (A) are present in Fig 1D. Plotted data are mean +/- standard deviation computed from three independent experiments. *P<0.05 (Student’s t-test). (C) Cell proliferation of NIH3T3 and NIH-RAS cells under conditions indicated. (D) Qualitative evaluation of ROS levels in NIH3T3 and NIH-RAS cells upon staining with DCFDA and analysis with a fluorescence microscope. (PDF) [file pone.0163790.s004.pdf]

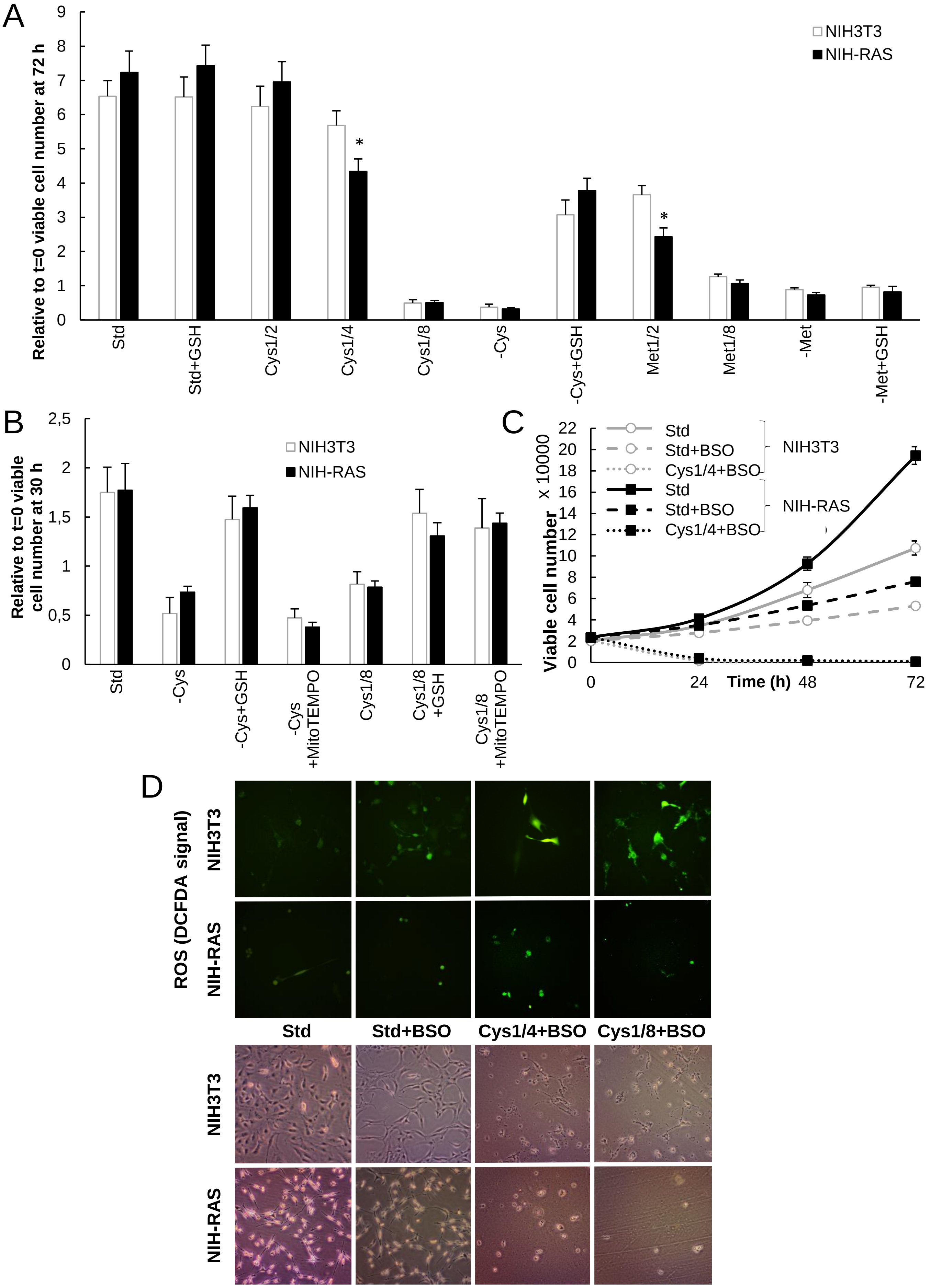

S4 Fig. Cell proliferation and qualitative ROS levels under different methionine concentrations and in cysteine-limiting or -depleted medium (possibly supplemented with antioxidants glutathione and MitoTEMPO or with GSH synthesis inhibitor BSO).
