# Supplementary material for: K-Ras Activation Induces Differential Sensitivity to Sulfur Amino Acid Limitation and Deprivation and to Oxidative and Anti-Oxidative Stress in Mouse Fibroblasts
Source: PLoS One. 2016 Sep 29;11(9):e0163790. doi: 10.1371/journal.pone.0163790 (PMC5042513; doi:10.1371/journal.pone.0163790)
Supplement: S5 Fig — Genome-wide transcriptional profiling datasets for NIH3T3 and NIH-RAS cells (available in NCBI GEO database; accession GSM741354-GSM741361 for NIH3T3 cells and GSM741368-GSM741375 for NIH-RAS cells), previously obtained in our laboratory with an MG_U74Av2 Affymetrix Gene Chip [41], were filtered for all genes encoding for solute carriers. Then, to identify genes whose expression was significantly altered in Ras-transformed versus normal cells (here represented in bold), a two-fold and a <0.05 cut-offs on Fold Changes and on p-values were used, respectively. In this Figure are represented all transporter genes with a fold change ≥2 (about 20% of all transporter genes) irrespective of their p-values. Gene Ontology (GO) enrichment based on molecular function was performed with GoTermFinder (http://go.princeton.edu/cgi-bin/GOTermFinder) and genes encoding for amino acid transporters were colored in magenta, while genes encoding for ion transporters were colored in grey. (PDF) [file pone.0163790.s005.pdf]

Gene symbol	P value	logFC	Gene title	Description
Slc6a15	**0.0371**	**-2.196**	**solute carrier family 6 (neurotransmitter transporter), member 15**	**Exhibits preference for the branched-chain amino acids, particularly leucine, valine and isoleucine, and methionine**
Slco3a1	**0.0383**	**-2.476**	**solute carrier organic anion transporter family, member 3a1**	**Mediates the sodium-independent transport of organic anions (such as estrone-3-sulfate), prostaglandins E1-E2, thyroxine, vasopressin**
Slc8b1	0.0505	-1.402	solute carrier family 8 (sodium/lithium/calcium exchanger), member B1	Mitochondrial sodium/calcium antiporter that mediates sodium-dependent calcium efflux from mitochondrion, thereby acting as a key regulator of mitochondrion calcium homeostasis
Slc25a15	0.0505	-1.269	solute carrier family 25 (mitochondrial carrier ornithine transporter), member 15	Ornithine transport across inner mitochondrial membrane, from the cytoplasm to the matrix
Slc29a1	0.0599	-2.822	solute carrier family 29 (nucleoside transporters), member 1	Mediates both influx and efflux of nucleosides across the membrane (equilibrative transporter) and is sodium-independent. It has a higher affinity for adenosine. Inhibited by dipyridamole and dilazep (anticancer chemotherapeutics drugs)
Slc7a2	0.0811	-5.343	solute carrier family 7 (cationic amino acid transporter, y+ system), member 2	Functions as permease involved in the transport of the cationic amino acids (Arg, Lys and Orn)
Slc9a3r1	0.0826	-1.968	solute carrier family 9 (sodium/hydrogen exchanger), member 3 regulator 1	Scaffold protein that connects plasma membrane proteins with members of the ezrin/moesin/radixin family and thereby helps to link them to the actin cytoskeleton
Slc35g1	0.1028	-2.412	solute carrier family 35, member G1	May play a role in intracellular calcium sensing and homeostasis
Slc39a8	0.1096	-1.478	solute carrier family 39 (metal ion transporter), member 8	Acts as a zinc-influx transporter
Slc35a5	0.1405	-2.343	solute carrier family 35, member A5	Probable UDP-sugar transporter
Slc38a4	0.1487	-1.890	solute carrier family 38, member 4	Sodium-dependent amino acid transporter. Has a broad specificity, with a preference for Ala, followed by His, Cys, Asn, Ser, Gly, Val, Thr, Gln and Met. May mediate sodium-independent transport of cationic amino acids, such as Arg and Lys
Slc38a9	0.1654	-1.207	solute carrier family 38, member 9	Lysosomal amino acid transporter involved in the activation of mTORC1 in response to amino acids
Slc44a1	0.1664	-1.338	solute carrier family 44, member 1	Choline transporter
Slc7a5	0.1756	-1.628	solute carrier family 7 (cationic amino acid transporter, y+ system), member 5	Sodium-independent, high-affinity transport of large neutral amino acids such as Phe, Tyr, Leu, Arg and Trp
Slc35e4	0.2068	-1.091	solute carrier family 35, member E4	Putative transporter
Slc7a1	0.3534	-1.017	solute carrier family 7 (cationic amino acid transporter, y+ system), member 1	High-affinity, low capacity permease involved in the transport of the cationic amino acids (Arg, Lys and Orn) in non-hepatic tissues
Slc9a9	0.3660	-2.264	solute carrier family 9 (sodium/hydrogen exchanger), member 9	May act in electroneutral exchange of protons for sodium across membranes
Slc25a35	0.3803	-1.070	solute carrier family 25, member 35	Mitochondrial carrier protein
Slc38a7	0.3926	-1.473	solute carrier family 38, member 7	Mediates sodium-dependent transport of amino acids, preferentially L-glutamine
Slc35d2	0.3929	-1.018	solute carrier family 35, member D2	Antiporter transporting nucleotide sugars pooled into the lumen of the Golgi in exchange for the corresponding nucleosides monophosphates
Slc46a3	0.4775	-1.230	solute carrier family 46, member 3	Orphan transporter
Slc6a9	0.5116	-1.223	solute carrier family 6 (neurotransmitter transporter, glycine), member 9	Terminates the action of glycine by its high affinity sodium-dependent reuptake into presynaptic terminals
Slc25a33	0.5341	-1.132	solute carrier family 25, member 33	Mitochondrial transporter that imports/exports pyrimidine nucleotides into and from mitochondria
Slc9a3r2	0.5552	-1.021	solute carrier family 9 (sodium/hydrogen exchanger), member 3 regulator 2	Scaffold protein that connects plasma membrane proteins with members of the ezrin/moesin/radixin family and thereby helps to link them to the actin cytoskeleton
Slc16a13	**0.0120**	**2.727**	**solute carrier family 16 (monocarboxylic acid transporters), member 13**	**Catalyzes the rapid transport across the plasma membrane of many monocarboxylates**
Slc43a3	**0.0463**	**2.110**	**solute carrier family 43, member 3**	**Orphan transporter**
Slc12a2	0.0555	1.217	solute carrier family 12, member 2	Electrically silent transporter system. Mediates sodium and chloride reabsorption. Plays a vital role in the regulation of ionic balance and cell volume

Gene symbol	P value	logFC	Gene title	Description
Slc14a1	0.0575	2.318	solute carrier family 14 (urea transporter), member 1	Urea channel that facilitates transmembrane urea transport down a concentration gradient
Slc6a8	0.0609	1.590	solute carrier family 6 (neurotransmitter transporter, creatine), member 8	Required for the uptake of creatine in muscles and brain
Slc4a4	0.1364	1.937	solute carrier family 4 (anion exchanger), member 4	Electrogenic sodium/bicarbonate cotransporter
Slc38a2	0.1366	1.178	solute carrier family 38, member 2	Functions as a sodium-dependent amino acid transporter. Mediates the saturable, pH-sensitive and electrogenic cotransport of neutral amino acids and sodium ions
Slc4a7	0.1407	1.634	solute carrier family 4, sodium bicarbonate cotransporter, member 7	Electroneutral sodium- and bicarbonate-dependent cotransporter
Slc12a7	0.1469	1.043	solute carrier family 12, member 7	Mediates electroneutral potassium-chloride cotransport when activated by cell swelling
Slc22a23	0.1703	1.773	solute carrier family 22, member 23	Orphan transporter
Slc25a44	0.2263	1.106	solute carrier family 25, member 44	Mitochondrial carrier protein
Slc5a3	0.3786	1.235	solute carrier family 5 (inositol transporters), member 3	Prevents intracellular accumulation of high concentrations of myo-inositol (an osmolyte) that result in impairment of cellular function
Slc2a13	0.6445	1.207	solute carrier family 2 (facilitated glucose transporter), member 13	Hydrogen-myo-inositol cotransporter

Blue: downregulated genes in NIH-RAS *vs* NIH3T3 cells; **Yellow**: upregulated genes in NIH-RAS *vs* NIH3T3 cells; **Magenta**: amino acid transporters; **Grey**: ion transporters

S5 Fig. Solute carriers differentially expressed between NIH3T3 and NIH-RAS cells.
