# Supplementary material for: K-Ras Activation Induces Differential Sensitivity to Sulfur Amino Acid Limitation and Deprivation and to Oxidative and Anti-Oxidative Stress in Mouse Fibroblasts
Source: PLoS One. 2016 Sep 29;11(9):e0163790. doi: 10.1371/journal.pone.0163790 (PMC5042513; doi:10.1371/journal.pone.0163790)
Supplement: S1 Table — Mass duplication times (MDT) for NIH3T3 and NIH-RAS under different methionine or cysteine concentrations (possibly supplemented with GSH) were calculated on semi-logarithmic curves represented in Fig 1A and 1B. Then, Student’s t-test was performed on linear regression curves for each nutritional condition that allowed cell growth. A = not parallel to linear regression curve of NIH-RAS cells in standard medium (99% CI); B = not parallel to linear regression curve of NIH3T3 cells in standard medium (99.9% CI); C = not parallel to linear regression curve of NIH-RAS cells in standard medium (99.9% CI); D = not parallel to linear regression curve of NIH3T3 cells in standard medium (99% CI); E = not parallel to linear regression curve of NIH3T3 cells in standard medium (99.9% CI). CI = confidence interval. (PDF) [file pone.0163790.s006.pdf]

	Mass duplication time (MDT) (h)		
**Growth condition**	**NIH3T3**	**NIH-RAS**	**Student's t-test on linear regression curves (NIH-RAS *vs* NIH3T3)**
Std	25	23	Parallel
Std+GSH	25	23	Parallel
Cys1/2	24	22	Parallel
Cys1/4	27	30[A]	Parallel
Cys1/8	No growth	No growth	-
-Cys	No growth	No growth	-
-Cys+GSH	38[B]	31[C]	Not parallel (99.9% IC)
Met1/2	38[D]	58[E]	Not parallel (95% IC)
Met1/8	No growth	No growth	-
-Met	No growth	No growth	-
-Met+GSH	No growth	No growth	-

S1 Table. Mass duplication times under different nutritional perturbations.
